# Retroperitoneal Lymph Nodes in Transitional Cell Carcinoma of the Kidney and Ureter

**DOI:** 10.1155/2009/181927

**Published:** 2009-01-26

**Authors:** Shilajit D. Kundu, Scott E. Eggener

**Affiliations:** ^1^Department of Surgery, Memorial Sloan-Kettering Cancer Center, New York, NY 10065, USA; ^2^Section of Urology, The University of Chicago Medical Center, Chicago, IL 60637, USA

## Abstract

The incidence of transitional cell carcinoma of the kidney and ureter is low and for that reason limited data exists regarding the appropriate management of regional retroperitoneal lymph nodes. Lymph node metastases have consistently been associated with an adverse prognosis. However, five-year cancer-specific survival following nephroureterectomy and lymphadenectomy for patients with lymph node involvement ranges from 0–39%, suggesting a therapeutic benefit. This review covers the primary tumor characteristics associated with lymph node involvement, imaging of the lymph nodes, as well as the rationale, role, patient selection, suggested anatomic templates, and technical considerations for lymphadenectomy.

## 1. Introduction

Transitional cell carcinoma (TCC) of the kidney and ureter, alternatively referred to as upper tract TCC (UT-TCC), represents approximately 10% of malignancies arising from the kidney and less than 5% of all urothelial malignancies, which primarily occur in the bladder. In 2007, about 3000 cases of UT-TCC were diagnosed in the United States compared to approximately 67 000 cases of bladder cancer [1, 2]. Within the upper urinary tract, TCC of the ureter is less common than TCC of the renal pelvis by a ratio of 1:4 [3]. There are a variety of treatment options available for UT-TCC including endoscopic excision or fulguration, segmental resection, and radical surgery. The management strategy selected primarily depends on the grade, stage, location, presence of multifocality, renal functional reserve, and the patient's comorbid conditions. Largely due to the infrequent disease incidence and variable lymph node templates, the role of lymphadenectomy for UT-TCC is not well defined. Since TCC of the bladder can be cured in approximately 25% of patients with regional nodal spread following an extended lymph node dissection (LND) and radical cystectomy, there is biologic plausibility to a therapeutic role for lymphadenectomy in patients with UT-TCC [4, 5]. This review will focus on the assessment and surgical treatment of lymph nodes in UT-TCC.

## 2. Relationship of Stage and Nodal Status with Outcome

Stage and grade of UT-TCC are independently associated with recurrence and survival. The five-year actuarial survival rates by primary tumor stage have been reported as 92%, 78%, 56%, and 0% for pathologic Ta-T1, T2, T3, and T4, respectively. Patients with stage T4 disease have a dismal median survival of 6 months [6, 7]. Tumor stage has consistently been shown to be the most powerful predictor of disease-specific survival [8–10]. However, other factors like higher grade, multifocality, lymphovascular invasion, and previous cystectomy have also been associated with inferior cancer-specific survival [10–12]. Transmural tumor growth (pT3 or pT4) is less common in distal ureteral tumors (33%) compared to midureteral (44%), proximal ureteral (75%), or renal pelvis tumors (41%). There are several plausible explanations for this observation. First, tumors in the proximal ureter may be less likely to cause obstructive symptoms compared to distal ureteral tumors due to greater distensibility of the proximal ureter and therefore present at more advanced stages than tumors in the distal ureter. Another proposed mechanism relates in part to the differences in muscular layers between the proximal and distal ureters. The distal ureter is encased by 3 layers of muscle in comparison to the proximal ureter which only contains 2 relatively thin interlacing layers [13]. This difference could explain the 2-fold higher incidence of transmural growth of proximally located tumors as compared to more distally located tumors [8].

Recent series show that up to 30% of patients with UT-TCC have regional nodal involvement [7, 14]. All the tumor characteristics that are associated with a poor prognosis are associated with an increasing likelihood of lymph node involvement. The likelihood of lymph node involvement is associated with increasing stage and ranges from 4% in noninvasive TCC of the upper tract to as high as 60% in patients with pT4 disease [10]. Hall et al. reported on 139 patients with pTa, pT1, or pCIS followed for a median of 64 months and not a single patient exhibited lymph node involvement at surgery or on follow-up [6]. Similarly, Kondo et al. reported on 42 patients with pTa, pT1, and pCIS, and there were no instances of lymph node metastases [14]. The five-year cancer-specific survival among patients with lymph node involvement varies widely and ranges from 0–39% [7, 14–17] (Table 1). Another study showed lymph node involvement to be independently associated with a three-fold increased risk of death at five years [7]. To our knowledge, no study definitively demonstrates a survival benefit for patients undergoing lymphadenectomy. Interpretations of studies including an LND are challenging for a number of reasons: (a) indications for an LND are not standardized, (b) templates are highly variable, (c) often only clinically suspicious lymph nodes are removed, and (d) in many series LND is applied for staging purposes and not therapeutic intent. Survival data from UT-TCC series is also confounded by 50% of patients having a history of bladder cancer and a significant number having cardiopulmonary morbidities, leading to competing risks of mortality [7]. However, Rabbani et al. compared Surveillance Epidemiology and End Results (SEER) outcomes for 657 patients who had UT-TCC diagnosed after bladder cancer to 7 839 patients who had de novo UT-TCC and found that patients with de novo UT-TCC had a 1.7-fold increased risk of cancer-related death [18]. Taken together, these data suggest that stage, grade, lymphovascular invasion, and tumor location are important factors that impact survival and should be addressed when considering treatment of UT-TCC and lymph node dissection.

## 3. Anatomic Distribution of Lymph Node Metastases

Anatomic lymph node mapping studies of the upper tract are rare due to multiple factors, including relative rarity of the disease, inconsistent dissection templates, and conflicting data on the role of lymphadenectomy in UT-CC. Tumors of the renal pelvis and upper ureter drain into the retroperitoneal lymph nodes, whereas tumors of the lower ureter drain predominantly into the pelvic lymph nodes. Work from Batata et al. in 1975 showed that node-positive tumors of the renal pelvis can involve upper retroperitoneal nodes (retrocrural, suprahilar, paracaval, paraaortic, and interaortocaval) and extend caudally to the external iliac lymph nodes [21]. Node-positive tumors of the middle and lower third of the ureter can also involve both the retroperitoneal and pelvic lymph nodes. Based on these findings, this group advocated an extensive LND encompassing both regions. Involvement of more than one lymph node has also been associated with an inferior 3-year cancer-specific survival compared to only one node involved (58% versus 16%) [22]. Kondo et al. recently reported the lymph node drainage sites of 42 patients based on pathologic or radiographic evidence of lymph node involvement. Tumors of the lower ureter demonstrated an approximately 10% rate of lymph node positivity, whereas tumors in the mid to upper ureters demonstrated an increased likelihood of positive lymph nodes (up to 42%). This was dependent on tumor stage and very few tumors that were pathologic T1 or less had lymph node involvement. Tumors of the right renal collecting system can metastasize to hilar, paracaval, retrocaval, interaortocaval lymph nodes, and right common iliac lymph nodes [23]. Rarely, in the setting of significant nodal disease, right-sided tumors will also metastasize to preaortic or paraaortic nodes. [14]. Tumors of the left renal collecting system may involve hilar, paraaortic, interaortocaval, and left common iliac lymph nodes [23]. Upper and midureteral tumors may also involve the same side-specific nodal regions as tumors of the renal pelvis, with midureteral tumors harboring the potential to spread to lymph node regions caudal to the inferior mesenteric artery extending along the common iliac nodal chain [14]. The nodal basins for tumors of the distal ureter include the external iliac, obturator, and hypogastric regions. Although constrained by limited lymph node dissections, these studies provide updated information on the lymph node drainage of the upper tracts in the current era.

## 4. Imaging of Lymph Nodes

Since staging of TCC plays an important role in dictating the course of therapy, accurate pretreatment imaging is of utmost importance. Computed tomography (CT) and magneticresonance (MR) imaging are the primary cross-sectional imaging modalities by which the regional lymph nodes are assessed. The rarity of UT-TCC has limited studies addressing imaging of these lymph nodes. However, imaging studies relating to TCC of the bladder can provide valuable insight. CT imaging can correctly stage lymph node involvement in over 70% of patients with muscle invasive bladder cancer. However, almost 25% of patients are understaged with clinically normal nodes on CT scan. CT has shown 28% sensitivity, 93% specificity, 68% positive predictive value, and 72% negative predictive value as relates to lymph node involvement in bladder cancer [24]. Jager et al. showed that MRI demonstrated a sensitivity, specificity, and accuracy of 83, 98, 92%, respectively, when lymph nodes were larger than 8–10 mm in patients with TCC of the bladder [25]. A common theme in most studies of CT or MRI in the staging of TCC is the relative understaging of lymph node involvement in radiographically “normal” nodes. The role of Fluorodeoxyglucose- (FDG-) positron emission tomography (PET) in the imaging of TCC of the urinary tract has been limited because it is excreted into the urine and hard to distinguish from tumor activity in the bladder or nearby lymph nodes. FDG-PET has helped to identify distant lymph node involvement and metastases for patients with TCC of the bladder but not well studied in UT-TCC [26]. However, with the advent and utilization of other radiotracer agents not excreted in the urine, such as ^11^C-choline, the future of PET imaging for UT-TCC is promising. ^11^C-choline PET-CT has shown encouraging preliminary data in detecting nodal spread of TCC of the bladder with pathologic confirmation of nodes as small as 5 mm [27]. While it seems intuitive that imaging studies of TCC of the bladder can be extrapolated to UT-TCC, this is not proven. Given the limitations of preoperative nodal imaging and the risk of understaging, LND remains the only way to definitively assess lymph node involvement in UT-TCC.

## 5. Role of Lymphadenectomy

As previously mentioned, sparse data exist to establish a well-defined role or optimal extent of LND. Virtually every study addressing LND for UT-TCC has been retrospective and severely limited by nonuniform application of LND, variable anatomic boundaries, and inconsistent selection for adjuvant therapy [6, 9, 14–16, 20, 28]. Therefore, determining whether LND provides a potential therapeutic benefit or simply offers more accurate surgical and pathologic staging is largely unknown. Nevertheless, the pertinent observations detailed above regarding lymph node metastases incidence, location, and prognostic significance have been made, subsequently reproduced, and help inform contemporary patient counseling and surgical management.

## 6. Staging

Intuitively, retroperitoneal LND should improve the accuracy of pathologic staging and allow for more accurate prognostic assessment. Therefore, complete surgical staging consists of a radical nephroureterectomy (NU) and regional LND. While no consensus has been established regarding the appropriate and necessary extent of a regional LND, multiple authors suggest patient-specific templates based on laterality of the primary tumor, location of the primary tumor (renal pelvis, upper/mid/lower ureter), and presence of radiographic or intraoperative retroperitoneal lymphadenopathy [14, 28, 29].

## 7. Therapeutic

While basic tenets of surgical oncology make it easy to proclaim that LND improves pathologic staging, it is largely unclear whether the time, effort, or potential morbidity of an LND is worthwhile from a therapeutic perspective. There are currently three lines of evidence suggesting that LND offers the potential for increasing the probability of cancer-specific survival. First, a proportion of patients with nodal metastases, up to 39%, exhibit intermediate-term cancer-free survival (Table 1) and highlight that regional nodal involvement is compatible with the possibility of durable cure. Second, multiple retrospective series, albeit with substantial limitations such as selection bias and the Will Rogers phenomenon [30], have shown that LND is associated with improved cancer-specific outcome [9, 16, 17, 28]. In a Japanese study by Kondo et al. of 169 patients with localized UT-TCC, a complete regional LND was associated with a 50% decreased risk cancer-specific death [28]. Roscigno et al. analyzed 132 patients undergoing NU for UT-TCC and five-year cancer-specific survival was 67% versus 40%, in favor of those having an LND [16]. Whether a patient underwent an LND in these series was surgeon determined and not prespecified. Therefore, factors such as patient age, clinical characteristics of the cancer, preoperative imaging features, comorbid diseases, and patient performance status undoubtedly influenced the decision of whether an LND was performed. We are unaware of any study that has randomized patients based on preoperative characteristics to inclusion or extent of LND. Until such a formal, prospective analysis is performed we will continue to debate the merits, value, and proper extent of an LND for UT-TCC. Third, since TCC of the UT and bladder originate from the same urothelial cells, it can be loosely extrapolated that patients with regional nodal involvement may respond similarly to surgical excision. Since the incidence of bladder TCC is much higher than UT-TCC, a richer experience with accompanying data has been established regarding the natural history of surgically treated node-positive patients. Approximately 25% of patients with bladder TCC and pelvic lymph node metastases will experience a durable recurrence-free survival [31], and up to 35–40% of patients with limited node involvement will experience low lymph node density or an organ-confined primary tumor [32, 33]. This attests to the curative potential of surgery for a subset of patients with lymph-node metastases in TCC of the bladder and suggests a similar possibility for those with node-positive UT-TCC.

## 8. Patient Selection

Without prospective studies utilizing standardized anatomic templates, the selection of patients for LND at time of treatment for UT-TCC remains uncertain. While other urologic malignancies such as prostate, bladder, and testicular cancer lack formal randomized data regarding the impact of LND, much has been gleaned from the outcomes of a uniformly applied, extensive LND consisting of similar anatomic boundaries for each patient [32, 34, 35]. This has not been done in a systematic manner for patients with UT-TCC, therefore decisions regarding the application or extent of LND must be drawn from weaker lines of evidence.

Among patients undergoing NU for UT-TCC, 16–23% will experience a local recurrence, typically in the regional lymph nodes [20, 36]. For patients with ureteral tumors, Park et al. reported a 37% versus 7% local recurrence rate based on the absence or presence of an LND, respectively [20]. Whether an LND may have prevented these recurrences, decreased progression to systemic metastases, or improved disease-free survival is not known. However, with local recurrence rates so high and their nearly universal association with subsequent metastases and disease-specific mortality, the benefit of an LND appears to outweigh the risks.

The incidence of nodal metastases has been strongly associated with primary tumor stage and multiple series have reported that lymph node metastases in the setting of low stage UT-TCC (≤ pT1) are rare at surgery or on follow-up [6, 14]. This data initially suggests that LND may be excluded for patients with low-stage disease however the gross limitations of preoperative clinical staging preclude an accurate and reliable prediction of ultimate pathologic stage. Therefore, it is our belief that all patients undergoing treatment for UT-TCC regardless of surgical approach (laparoscopic or open) or type of surgery (segmental ureterectomy, partial nephrectomy, or radical NU) should have a concomitantly thorough regional LND.

## 9. Technical Considerations for Lymph Node Dissection

As UT-TCC may originate from either the renal or ureteral urothelium, the regional lymph nodes may vary and the anatomic regions of LND should be planned accordingly.

Based on the regional anatomic drainage detailed above and presuming that the surgeon prefers an extensive rather than a suboptimal LND, recommendations regarding LND anatomic boundaries can be offered. The anatomic borders of dissection for a left-sided renal pelvis, upper ureteral, or midureteral tumor should, at the minimum, encompass the paraaortic, preaortic, and interaortocaval nodes from the level of the renal hilum to the aortic bifurcation. For a right-sided renal pelvis, upper ureteral, or midureteral tumor the removed node regions should include the paracaval, precaval, and interaortocaval areas from the renal hilum to the aortic bifurcation. For most midureteral tumors and all distal ureteral tumors, regardless of side of origin, a common iliac, external iliac, obturator, and hypogastric lymphadenectomy should be performed.

The method of LND, either open or laparoscopic, is a secondary concern compared to the primary intent, which is a thorough and safe removal of regional lymph nodes. Between 43–72% of patients undergoing open NU have a simultaneous LND and it is our impression that patients undergoing laparoscopic NU do so even less frequently [15, 16, 20, 28]. Busby et al. have recently compared patients undergoing open and laparoscopic NU with LND and noted a slightly higher lymph node yield for patients undergoing the laparoscopic approach (median: 6 versus 3) [37]. The authors do not state what proportion of patients requiring NU had an LND. Both groups appear to have an inadequately low yield and surgeon template preference can easily, and likely did, account for the differences rather than surgical approach.

The surgeon's goals are to optimize pathologic staging, minimize local recurrences, and potentially improve disease-free survival. General technical concerns for retroperitoneal LND include “split and roll” of the inferior vena cava and/or aorta, meticulous hemostasis and lymphostasis, and readiness to control and ligate lumbar vessels when necessary. For left-sided templates, the left renal vein is positioned as the superior border unless suprahilar adenopathy is present, in such case the surgeon should consider a more cranial dissection. If bulky retroperitoneal metastases are bilateral and resectable, ejaculatory function may be of concern. However, since the median age at diagnosis of UT-TCC is typically in the late 70s [38], this situation should be rare. Since the sympathetic trunks, ganglia, and postsympathetic efferents are collectively responsible for antegrade ejaculation, their preservation, either by nerve-sparing or a modified template dissection [39], is essential to maintain normal ejaculatory function.

## 10. Future

Given the aforementioned limitations in retroperitoneal imaging, preoperative staging, and the devastating impact of local recurrences, we feel that an LND using a standardized template should be considered for all patients undergoing NU. However, if more accurate imaging with novel modalities such as lymphotrophic tracers, molecular agents, or PET scans becomes available, the treatment paradigm for patients with UT-TCC would be appropriately altered.

Neoadjuvant chemotherapy for patients with muscle-invasive bladder TCC results in improved disease-specific outcomes [40] and, given the parallels between bladder and UT-TCC, may be ideally suited for patients with high-risk UT-TCC. For UT-TCC, neoadjuvant chemotherapy has not been adequately studied and may never, given its relative rarity. Beside the potential for improving cancer control, other benefits may include optimizing renal function at the time of administration, allowing for maximum doses of the most active agents, and eliminating the possibility of surgical complications delaying adjuvant therapy. Since the gemcitabine and cisplatin doublet provides similar efficacy to traditional MVAC (methotrexate, vinblastine, doxorubicin, and cisplatin) regimens in patients with bladder TCC, with an improved safety profile, neoadjuvant therapy for patients with UT-TCC becomes even more attractive [41].

## Figures and Tables

**Table 1:** Five-year cancer-specific survival for node-positive disease following nephroureterectomy and lymph node dissection.

	Number of node-positive patients	Five-year cancer-specific survival
Johansson and Wahlqvist [19]	N/A	0%
Secin et al. [15]	28	0%
Miyake et al. [17]	13	0%
Novara et al. [7]	27	12%
Kondo et al. [14]	42	15%
Park et al. [20]	11	27%
Roscigno et al. [16]	26	39%
